# Healthcare professional perspectives on medication challenges in the post-stroke patient

**DOI:** 10.3389/fphar.2023.1266277

**Published:** 2023-11-21

**Authors:** Shauna Bell, Helen Kelly, Eva Hennessy, Margaret Bermingham, Jennifer Raymy O’Flynn, Laura J. Sahm

**Affiliations:** ^1^ Department of Speech and Hearing Sciences, University College Cork, Cork, Ireland; ^2^ School of Pharmacy, University College Cork, Cork, Ireland

**Keywords:** stroke, medication adherence, continuity of care, healthcare professionals, pharmacy, speech and language therapy, Occupational therapy

## Abstract

**Background:** Medications play an essential role in the management of patients who have experienced a stroke. Despite the recognised importance and widespread availability of secondary prevention guidelines, Irish research has shown a continuous failure to meet secondary prevention targets upon discharge. While complex interventions involving healthcare professionals (HCPs) such as Speech and Language Therapists (SLT), Occupational Therapists (OTs) and Pharmacists have been effective in combatting medication non-adherence, community multidisciplinary teams (MDTs) are not as well defined as in the acute setting, leading to wide variation in patient care. Therefore, this study aims to investigate the knowledge, attitudes, beliefs, and challenges faced by HCPs in the continuity of care post-discharge from a hospital stroke ward, and its impact on medication adherence.

**Methods:** Semi-structured interviews and one focus group with HCPs were conducted, and data were analysed using Braun & Clarke’s reflexive Thematic Analysis.

**Results:** Fourteen HCPs (6 Pharmacy, 4 SLT, 4 OTs) participated in this study. Participants discussed their views under two main themes 1) continuity of care and 2) medication adherence. Sub-themes observed regarding continuity of care include management and organisation, interpersonal continuity, and informational continuity. Themes generated which impact medication adherence post-discharge include condition-related factors, medication-related factors, systemic and HCP factors, and patient-related factors.

**Discussion:** Additional resources are required to bring community healthcare in line with the standard of acute care. Increased channels of communication must be established across contexts and disciplines, and may be achieved using interprofessional training through continuous professional development or third-level education, a more clearly defined community team structure, and discharge summaries completed to relevant quality standards. While suboptimal continuity of care was reported as contributing to medication non-adherence, HCPs also acknowledged the complexities of medication management post-stroke.

## 1 Introduction

Approximately 7,500 people in Ireland are diagnosed with stroke each year, which has been identified as the leading contributor to adult acquired physical and neurological disabilities (Health Service Executive, 2022). One of the most prominent risk factors for stroke is the presence of previous stroke or transient ischemic attack, where nearly one in four diagnosed cases are classed as a recurrent cerebrovascular accident (CVA) (Tsao et al., 2023). Risk of recurrent stroke has been found to rise to between 30% and 43% within 5 years of the initial cerebrovascular event (Chambers et al., 2010) and incidence of recurrence or death post-stroke rises to 67.7% within 10 years of initial stroke (Flach et al., 2020).

Medications play an essential role in the management and treatment of patients who have experienced a CVA, and substantially decrease the risk of recurrent stroke (Hackam and Spence, 2007). Clinical guidelines recommend several secondary prevention antihypertensive and lipid-lowering medications which should be started immediately and continued indefinitely following the cerebrovascular event (Intercollegiate Stroke Working Party, 2023). While these medications have been specifically prescribed to improve the health and wellbeing of the patient, their positive effects are often hindered by the fact that an estimated 50% of patients are reported not to take medications as directed (Brown and Bussell, 2011). Whether intentional or non-intentional, medication non-adherence is associated with almost 200,000 deaths annually (van Boven et al., 2021). From an economic perspective, non-adherence is responsible for €80–125 billion of potentially preventable direct and indirect costs in the EU (van Boven et al., 2021). The identification of these factors has led to adherence to pharmacotherapy being pinpointed as the most significant long-term target of medical management of stroke (Smith et al., 2012; Dalli et al., 2021).

Despite the recognised importance and widespread availability of secondary prevention guidelines, research conducted on an Irish population has shown a continuous failure to meet secondary prevention targets (Brewer et al., 2015). This is due in part to the fact that in the post-stroke period, patients face a multitude of medication-related challenges. These challenges are poorly characterised in the literature, especially at the transition of care between hospital and home (Andrew et al., 2018). This is particularly true of the experiences of those with more severe stroke-related impairments, who are often excluded from explorations of medication adherence. Though these patients have the capacity to meaningfully participate in healthcare research, their physical, communicative, and cognitive needs often result in their exclusion (De Simoni et al., 2015).

Early, efficient community-based stroke rehabilitation and disability management must be offered to all stroke patients leaving hospitals who require it through a dedicated multidisciplinary team structure (Intercollegiate Stroke Working Party, 2023). Physiotherapy, Occupational Therapy (OT) and Speech and Language Therapy (SLT) are the only disciplines currently reporting into the stroke register regarding access to therapy supports in acute stroke units in Ireland (Health Service Executive, 2022). The majority of these patients have ongoing therapy needs after the acute phase, with the highest demand (52%) being placed on continuing SLT services (Health Service Executive, 2022). However, the broader multidisciplinary team (MDT) structure is ill-defined, with its members dependent on context (e.g. acute care, community care) or purpose (e.g. rehabilitation, palliation). In particular, the concept of MDTs in community settings are not as well defined as they are in the acute setting, leading to wide variation in those involved in the patient’s care (Weiss et al., 2014). This is significant as approximately 60% of patients are discharged directly to home following the acute event and rely on community services for their rehabilitation (Health Service Executive, 2022). Depending on stroke severity and availability of resources, patients may also be discharged to complex specialist rehabilitation units, or long-term care facilities (Health Service Executive, 2022). It is widely acknowledged that rehabilitation services are under-developed in Ireland, with the Irish National Stroke Strategy 2022–2027 placing emphasis on the development of organised stroke pathways (Health Service Executive, 2022). Only one-fifth of sites have access to Early Supported Discharge (ESD) teams, a feature of Irish stroke pathways which provide specialist rehabilitation in the community to facilitate an accelerated discharge from the acute setting (Collins et al., 2021). Five of the nine ESD programmes in Ireland are situated in the country’s capital with only one hospital serving rural dwellers (Health Service Executive, 2022). These ESD services highlight the importance of team composition and multidisciplinary co-ordination in delivering standard-meeting services (Chouliara et al., 2023), and clearly outline the inclusion of OT, SLT, Physiotherapy, Medical Social Worker, Clinical Nurse Specialist and Therapy Assistant as central roles (Health Service Executive, 2022). However, it is acknowledged that other HCPs also play a key role in providing targeted community rehabilitation for stroke survivors, such as Pharmacy, Dietetics and Psychology (Health Service Executive, 2022).

Continuity of care is considered an important determinant of medication adherence (Yao et al., 2022). While complex interventions involving healthcare professionals have been effective in combatting medication non-adherence (Simoni et al., 2009; Chung et al., 2011) research must be conducted to determine specific roles and tasks within the team for a seamless transition of care (Nieuwlaat et al., 2014). The first step in this process is to establish the current patterns of care from the perspectives of HCPs, and its perceived shortcomings (Hill et al., 2009). As healthcare systems, procedures, and beliefs vary from country to country, research must reflect the national healthcare landscape (Bauler et al., 2014) and importantly include the perspectives of the HCPs who provide this care. Pharmacists, Speech and Language Therapists and Occupational Therapists all play a role in medication management post-stroke (Health Service Executive, 2022). The Pharmacist is central to all aspects of medication-taking post stroke, most notably dispensing and managing pharmacotherapies (Al-Qahtani et al., 2022). Speech and Language Therapists assess and evaluate the ability of the patient to safely swallow medications (Brown and Bussell, 2011). In addition, they collaborate with the MDT to ensure health information is accessible and appropriate for patients’ communicative needs (Brown and Bussell, 2011; Health Service Executive, 2022). The Occupational Therapist is responsible for assisting patients to engage in meaningful and purposeful Activities of Daily Living (Brown and Bussell, 2011). Medication management has been flagged as an ADL essential for allowing an individual to live independently in the community (Allen et al., 2023). Therefore, this study aims to investigate the knowledge, attitudes, beliefs, and challenges faced by HCPs (specifically, Pharmacists, Occupational Therapists and Speech and Language Therapists) in the continuity of care post-discharge from a hospital stroke ward, and its impact on medication adherence.

## 2 Methods

### 2.1 Recruitment

This study received ethical approval from the Clinical Research Ethics Sub-committee (CREC) at University College Cork. A purposeful sample of Pharmacists, SLTs and OTs were recruited via email and word-of-mouth. Participants were considered eligible if they were currently practising in the fields of Pharmacy, SLT or OT.

### 2.2 Data collection

This study had a phenomenological underpinning. Phenomenology is often used in explorations of healthcare professional’s perspectives, as it gives a unique insight into the participants lived experience of a phenomenon, while also acknowledging the existing literature. Semi-structured interviews and focus groups with HCPs were conducted by SB, with EH observing and taking field notes. Eleven individual interviews were conducted online. Mean interview time was 25 min 46 s, with a range of 10 min 46 s to 48 min 02 s. In addition, one focus group with three Occupational Therapists was carried out. Focus group time was 49 min 39 s. These semi-structured interviews were conducted in-line with a pre-established topic guide (Supplementary Appendix I), where questions were generated based on the World Health Organisation (WHO) Framework on continuity and coordination of care in integrated people-centred health services (World Health Organization, 2018). Participants were asked to consider each question in terms of medication-related information they and/or the patient may receive. Open-ended questions relating to barriers and facilitators to medication adherence post-stroke were also asked in order to capture salient ideas unrelated to continuity and co-ordination of care (Weller et al., 2018). Interviews were conducted via a closed channel on Microsoft Teams, where each participant was provided with a unique meeting code to ensure data protection. Written informed consent was obtained prior to the meeting and was also recorded verbally at the start of the interview.

### 2.3 Data analysis

Data analysis was conducted in line with Braun & Clarke’s reflexive Thematic Analysis (Braun and Clarke, 2019) as previous research with HCPs has found this appropriate for investigating knowledge, attitudes, and beliefs of participants (Jaam et al., 2018; Kvarnström et al., 2018). Semi-structured questions facilitated inductive generation of themes through the extraction of meaning and identification of trends from data (Galletta, 2013). Themes were not pre-specified prior to analysis. A six-step protocol was followed by SB and EH:

1. Familiarisation with the data: Interview recordings were divided amongst SB and EH for transcription. Both SB and EH engaged in a process of immersion in the data through the thorough examination and re-reading of transcripts. They maintained individual notes on the content and contextual nuances of the data for discussion with the research team.
2. Generating codes: Significant elements of the data were methodically and systematically identified and labelled. SB and EH conducted this step independently. Both researchers then came together to organise these codes into broader categories to represent ideas pertinent to the study issue.
3. Generating themes: SB and EH conducted a systematic exploration of patterns evident in the coded data. This allowed the researchers to generate themes which represent the relationships between several different codes.
4. Reviewing the themes: Critical examination and refinement of the identified themes took place in this stage where SB and EH ensured that the themes were coherent, meaningful, and accurately represented the data.
5. Defining themes: The boundaries and specific characteristics of the themes were clarified, ensuring they accurately captured participants' experiences or perspectives.
6. Write-up: a coherent and comprehensive narrative was composed that presents the research findings based on the identified themes.

## 3 Results

### 3.1 Participants

Fourteen HCPs (1 Male: 13 Female), participated in this study (Table 1). Of the 14 participants, six were Pharmacists, four were Speech and Language Therapists, and four were Occupational Therapists. Both junior and senior roles were represented within these professional groups. Median years of practice was 10 years, with an interquartile range of 0.83 years–25 years.

**TABLE 1:** Participant demographics.

Participant code	Role	Setting	Years of practice
HCPP1	Locum Pharmacist	Community	11
HCPP2	Pharmacist	Community	10
HCPS3	Speech and Language Therapist	Acute/Community	2
HCPP4	Supervising Pharmacist	Community	5
HCPP5	Superintendent Pharmacist	Community	19
HCPP6	Supervising Pharmacist	Community	25
HCPP7	Pharmacist	Community	3
HCPS8	Senior Speech and Language Therapist	Acute	22
HCPS9	Speech and Language Therapist	Community	2
HCPO10	Occupational Therapist	Community	0.83
HCPS11	Senior Speech and Language Therapist	Acute	13
HCPO12	Occupational Therapist	Community	2
HCPO13	Occupational Therapist	Acute	2
HCPO14	Occupational Therapist	Acute	2

### 3.2 Themes and Subthemes

**FIGURE 1:**
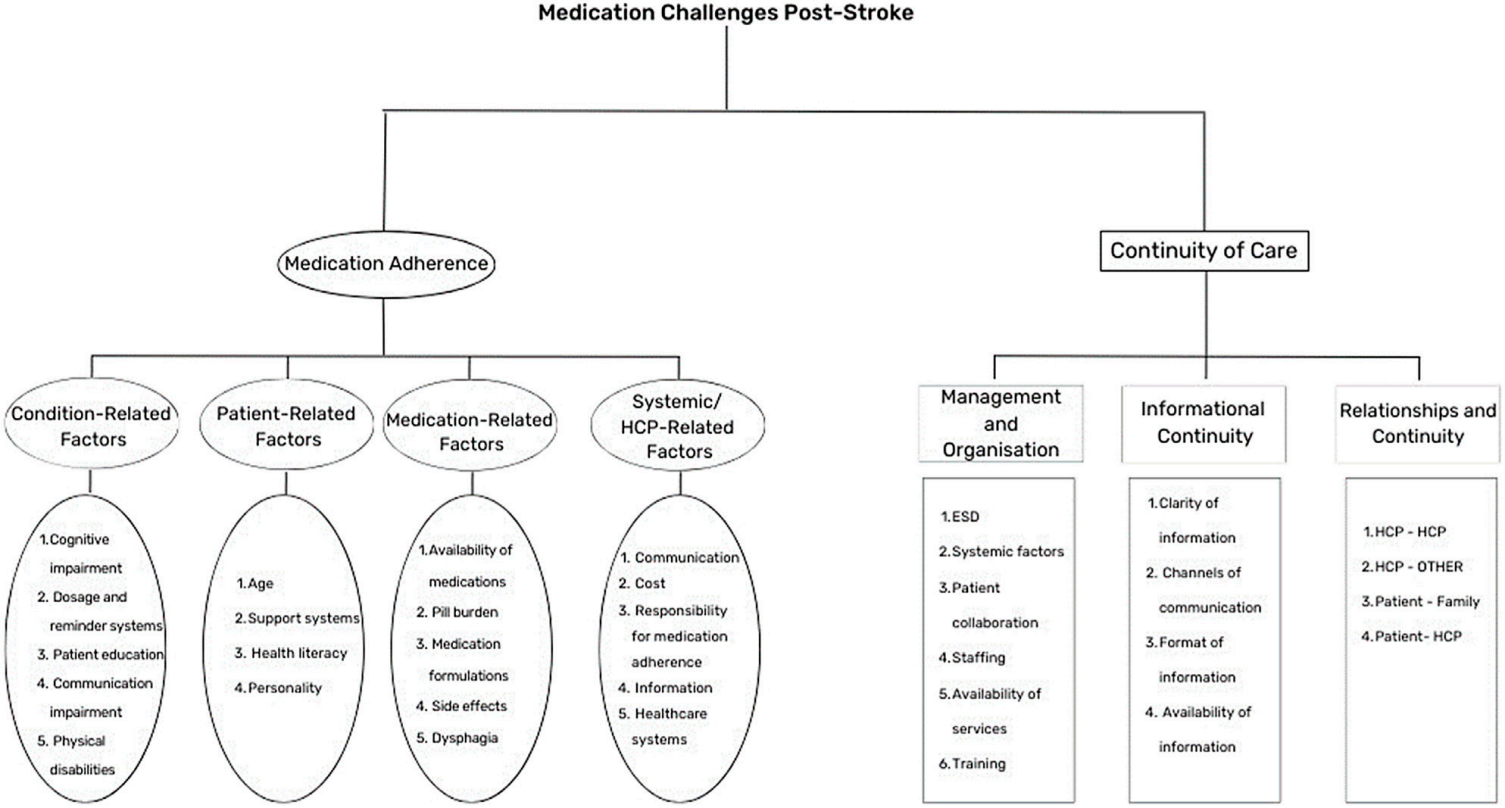
Themes and Subthemes identified.

The following themes were observed (Figure 1):

(i) Medication Adherence. This theme explored elements which may impact medication taking behaviours of the patient. Four sub-themes were observed: condition-related factors, medication-related factors, systemic factors, and patient-related factors.
(ii) Continuity of Care. This theme focused on the ongoing experience of the patient as they progress through different parts of the service and interact with different members of the healthcare team. This encompassed three sub-themes: management and organisation, interpersonal continuity, and informational continuity.

### 3.3 Theme 1: medication adherence

The theme of Medication Adherence encompasses the complex factors influencing an individual’s ability and willingness to consistently follow prescribed medication regimens, including challenges and strategies associated with adhering to treatment plans.

#### 3.3.1 Sub-theme: condition-related factors

All participants reported that the occurrence of a stroke could increase medication non-adherence. In particular “if they’ve got language or cognitive issues, that’s going to make it difficult for them to independently manage medication” (HCPS8). Features of cognitive and communicative impairment which participants considered most directly impact medication adherence include “poor recall” (HCPO14), “issues with understanding what their medicines are for and how to take their medicines” (HCPP1), and “fatigue” (HCPS8). Participants stated that the impact of these aspects on medication adherence could be addressed through patient education, though HCPP4 stressed that this should be provided in a manner that doesn’t “overload them or overbear them”. Participants voiced different views on how this patient education should be provided. Some preferred “more tangible, permanent communication” (HCPS8) such as “a leaflet” (HCPP5), “picture charts … having a picture or the actual tablet stuck on” (HCPS8), or a “fact file” with “abbreviations or [drug names]” (HCPS9). HCPP2 indicated a preference for providing information “orally … people don’t really read stuff … and then you can ask them if they have any questions”. HCPS8 noted that this may be useful for those with alexia (acquired dyslexia/reading difficulties), though felt that “an overall total communication approach” should be taken so “people can go back to it … even if they can understand the verbal speech, if you have emotional or … vulnerable factors in it, you’re not going to be taking it in”.

Eight participants highlighted the benefits of using medication dosage systems to overcome linguistic and cognitive deficits. The systems most mentioned by participants included “dosette boxes” (HCPS11) or “weekly or daily organisers” (HCPP1) and “blister packs” (HCPP5). Participants also found reminder systems to be helpful, such as “reminders on your phone … a notebook or the communication diary”, “checklists” (HCPS9), “apps” (HCPS3), “[pillboxes] that will alarm” (HCPO10), and “a text message service” (HCPP4). Participants acknowledged that these are not without their drawbacks, with HCPS8 reflecting “you do out like this typed list, and then you know if something changes you have to do a new typed list, then they lose the list”. HCPP5 also found that with blister packing “sometimes it only causes more confusion”, particularly for those with physical disabilities post-stroke: “the things like dexterity—they might have been able to open blister packs before, they can’t afterwards”. However, HCPO13 felt that dosette boxes and blister packs might be a facilitator to medication adherence for those with impaired dexterity.

#### 3.3.2 Sub-theme: patient-related factors

The majority of participants highlighted the impact that individual patient factors may have on medication adherence post-discharge. In particular, “the personality of the person, whether they want to take it or not” (HCPS9) was seen as an important predictor of adherence. Participants felt that those with lower health literacy were less likely to adhere to medications as “sometimes people can’t follow what you think is a basic instruction, so health literacy [is a barrier]” (HCPP2). While participants acknowledged that “If there’s any cognitive impairment or issues with understanding what their medicines are for … that would be a barrier [to health literacy]”, others found that “they weren’t managing their medication before they had their stroke” (HCPO10). HCPP2 stated that some patients have no desire to increase their knowledge: “sometimes they don’t want to know … sometimes they have no interest. They just are like I was told to take this so I’ll take it … It’s scary how happy they are just to take what’s prescribed sometimes” (HCPP2). Participants highlighted that older patients may need more support and education around their medications as “younger patients, they would know and they”d say, “oh, I need my blood thinner” or “I need my blood pressure”. Older patients wouldn’t and they would maybe go more for “I need the small yellow tablet I’ve ran out of those” (HCPP4). HCPO10 acknowledged that while many patients dislike taking multiple medications, older patients may be particularly averse to polypharmacy: “They just don’t like being on tablets, because it’s like, oh, I’m old if I take all these tablets”.

One patient-related factor proposed by all participants was the presence of a support system for the patient post-discharge. Carers were seen to be an invaluable asset for medication management: “You’re very reliant on family, when it comes to it, so. If somebody can’t manage their own medication, and they don’t have a reliable family member available, that’s really going to impact discharge” (HCPS11). Pharmacists such as HCPP6 stated that “generally it's never the patient I’m dealing with it’s a family member. You know, they don’t tend to come in. Obviously, there’s different stages, there’s different disabilities, post stroke … But generally speaking, they’re so overwhelmed by the change in their life that their input is minimal”.

#### 3.3.3 Sub-theme: medication-related factors

Participants considered pill burden, formulation and availability of medication, and side-effects to be the greatest barriers to medication adherence in this cohort. HCPP1 noted that “the fact that some of these patients may have started on no medicines and now they have quite a high pill burden can be a barrier to adherence,” while HCPP7 also commented on the “complexity of regimes.” HCPO14 saw it as the role of the Occupational Therapist to consider “if they need to take their meds twice a day, and we don’t think that’s going to happen, have we put in support two times a day?“.

Both Pharmacists and Speech and Language Therapists considered the impact of dysphagia (impaired swallow) on medication adherence. Participants felt that “if they have swallow issues, that’s going to make things difficult … sometimes you have to work around it” (HCPS8). Participants noted that this could be addressed in most cases by “chang [ing] the formulation of their tablets” (HCPP6) or adjusting based on clinical judgements (HCPS8): “if I’m saying a patient’s nil per oral but there’s some drugs that they particularly need … you have to give it … [the] benefit of that outweighs the risk of them aspirating”. HCPP6 noted that “We mightn’t know that they don’t like taking their medication because it's uncomfortable for them to take, or they can’t physically take it”. HCPS8 noted that while the role of the Speech and Language Therapist is to assess swallow function, “we don’t make recommendations as to what form patients should take their medications in … there’s kind of a standard line on the cover sheet of [the discharge pack] going: “if you have any queries about taking your medications, liaise with your pharmacy … if I put the patient on modified diet and fluids and I don’t think they can manage their medications whole, I’ll send a referral to pharmacy”. HCPS3 found that it may be difficult for the patient to advocate for their swallowing needs, as “a patient, if they have a swallowing difficulty and aphasia, they can’t necessarily tell you “I”m supposed to be having thickener in my tea”. As a Pharmacist, HCPP1 reported that this information was not easily accessible to them, and “the only way sometimes that I can tell … they have issues with, say swallowing, for example, is if they were prescribed a thickener. II So it’s not always clear that that may be present in a patient”. This was not seen as the only barrier to providing alternative drug formulations. HCPP5 described sourcing liquids as being “kind of difficult”, as “liquid formulations aren't generally covered on the HSE schemes. So you’re looking at crushing tablets, that side of it can be awkward”.

Where appropriate medications and formulations have been prescribed, participants found that patients may discontinue medications due to side effects. In HCPS9’s experience, “they won't take a certain drug 1 day because they’re like, I don’t need that one, or I don’t like the side effects that one gives me. And I feel like I get I feel more nauseous when I get this one or I feel more down, or I feel more out of sorts, more tired when I take that one”. HCPO12 found that the potential to experience drug side effects has led patients to “withdraw from drugs and go into alternative therapies. And then it just cyclical, it just comes back around”. The most common side effects mentioned by participants were “dry mouth” (HCPS3), “reflux” (HCPS9), “nausea” and “fatigue” (HCPO10).

#### 3.3.4 Sub-theme: systemic/HCP related factors

The largest systemic contributor to medication non-adherence was, in the words of participants, “breakdown in communication or lack of continuous communication” (HCPP7). HCPP1 stressed the importance of achieving “continuity of medicines, so that the discharge prescription is accurate, that the patient has been started on any medicines that should be newly started, that any medicines that should have been stopped are stopped” to facilitate optimum adherence.

While all participants mentioned that medication non-adherence should be addressed, there was discrepancy among participants regarding which HCPs be involved. Pharmacists were the HCP most commonly associated with medication management, and were seen to have a role in prescribing, patient education, and measuring adherence. GPs were also seen to have a large role in prescribing, counselling, and monitoring medication usage as “their communication of the importance of adherence to the particular meds and stuff like that can be very well received by patients” (HCPP7). Public Health Nurses were recognised as playing an important role in medication adherence, as often “nurses spend more time with the patients” (HCPS3). The role of the Speech and Language Therapist was less recognised, though Pharmacy participants noted their role in “formulations, and what formulations are suitable” (HCPP1), “the thickening agents and such” (HCPP6). Only two participants noted the role of the Occupational Therapist, which would occur if “they can’t physically hold something or they need an easier way to manage something” (HCPP6). HCPS9 reported seeing medication management as involving “to an extent, everyone in the MDT including the patient and the family members … some roles definitely bigger than others”.

Some participants felt that these roles should clearly be addressed in clinician training. HCPS9 observed “could it be promoted to a bigger extent? Yeah, probably. And I think that would also come from probably education and college … to clarify what your role is, but also how we all have a role to play on, say, for instance, medication, having that knowledge kind of drilled into every clinician from education up”. HCPS3 felt that this lack of training is evident in current practice: “I did a training with some new starter physio and Ots recently … their knowledge was really limited and through no fault of their own, but they just hadn’t been equipped with those resources”. HCPP7 thought that resources in this area should be available to practicing HCPs: “I would probably feel additional resources, support or information would definitely help maximize my input into the patients holistically”. HCPS11 felt that current knowledge gap may impact patients’ healthcare experiences: “the information that’s provided isn’t enough, and I don’t have the answer for them when they ask”. However, some sites are taking their own measures to address staff knowledge gaps: “In my last rotation, we did like a drug of the week. So one person from the team would go pick a drug … they’d go off and research it and then just give a few minutes chat through what it is, what it’s used for what are the side effects … you might not always get the information from the doctors, so it’s a good way to go about like getting the knowledge yourself” (HCPO13).

Regardless of the HCP administering medication counselling, all participants regarded patient education as paramount to medication adherence. However, HCPP2 noted that the current healthcare system does not allow the time needed to successfully carry this out: “it’s kind of the Pharmacist’s job just to try and explain about what the different things are, what they’re for, why they’re important. But we always don’t—we don’t always have time to do that because we also have to do 10 other things at the same time”. They proposed that “a systematic approach that everybody followed all the time and there was enough people working to be able to do it” would successfully address this gap.

Four participants viewed cost as a barrier to medication adherence. HCPS3 noted that “some people aren’t on drugs schemes. A big prescription can cost an awful lot of money”. HCPO14 also addressed the fact that “if you’re, you’re not on a medical card … you’d rack up a hefty bill quite quickly”. Even for those eligible for drug payment schemes (HSE.ie, 2023), “it’s capped at €80 [per month] but it’s still a lot of money. That we’d would try and help people to set that up, but it only lasts for 3 months” (HCPP4). In HCPS8’s experience “a lot of people here would end up being eligible for the medical card (HSE.ie, 2023) if they have reason. So I don’t think the cost of medication day-to-day necessarily. But like the cost of providing … you know, there’s very few people probably could pay for private carers for a nurse to come in to actually supervise someone taking their medications”. Even for those with the funds to “access home help to supervise medication administration, they’re not actually allowed touch the medications … if they come to me and I put out two of my tablets instead of one, I don’t know what they’re actually allowed to do” (HCPS8).

### 3.4 Theme 2: continuity of care

This theme, encompasses HCP experiences and perceptions of patient access to healthcare services as a whole over time, and relates to acute and community services in both private and public settings. Each participant was asked to provide a definition of their understanding of continuity of care. Most referred to continuity of care being “a good handover of information from one HCP to another” (HCPP2), particularly with regard to “the transition of patients from one section of society to another” (HCPP6). HCPO10 viewed continuity of care as the “gold standard” of hospital discharge, whereas others (HCPO13, HCPO14) indicated that while the principles of continuity of care were familiar to them, they “hadn’t really heard of the term before”. Five participants discussed the importance of having “the same level of care given all the time” particularly as “people’s needs change as they progress” (HCPP4). HCPS9 stressed the importance of recognising the roles of “professionals, family members and the client” in the transition of care.

#### 3.4.1 Sub-theme: management and organisation

Almost half of the participants viewed staffing shortages as a barrier to efficient continuity of care, as “sometimes there just isn't the option to offer that continuing care” (HCPS3). HCPs felt that this impacted their ability to support their patients, as “there’s not enough staff trying to do all the jobs that are needed to be done, to make sure that it's done seamlessly” (HCPP2), even though “the people on the ground that are doing it, are really trying their best” (HCPO10). This is particularly true “on discharge day,” where HCPP2 felt that more staff was needed in order for them to be “contactable” by community HCPs. HCPP7 compared the staffing levels within the Irish system to the National Health Service (NHS) in the UK and felt that “important steps of discharge can be missed just because of the infrastructure of Irish hospitals”. Other participants felt that issue was also prevalent in the community, which is reflected by “the waiting list. They’re so long” (HCPO10).

HCPS3 felt that lengthy waiting lists for publicly-funded therapy services reflected the lack of individualised pathways for stroke patients, as HCPs are “dealing with the outpatient community outside of people who went through, say, the stroke pathway”, which leads to them “missing out on all that recovery and support, especially within the six first months of all that spontaneous recovery”. HCPS11 acknowledged that services were taking steps to address this gap, as “there’s people who are developing pathways and that’s high on the agenda”. Other factors which HCPS3 noted as barriers to service access for patients were their “postcode, funding, and social care”.

HCPP6 felt that there was a discrepancy between services available to those in acute pharmacy in contrast with those working in a community setting “In hospital pharmacy, I mean, I’d access to the NEWT guidelines, the handbook of enteral feeding. whereas in community you don’t tend to have those same resources. Now I know where to find them but not everybody does, because I’ve come from that background. But even the information I can find is quite outdated”.

One service which participants felt worked well with regard to continuity of care for the stroke patient was the Early Supported Discharge (ESD) team. The core team members of “a speech therapist, a physio, and an OT” (HCPS11) described as providing “intense rehab” for those who are “medically fit and they don’t need to be in hospital” (HCPS8). The ESD service is seen to “enable patients to get out of hospital” while providing immediate support (HCPS8). ESD provides patients with “a familiar face … someone that knows your history, how you communicate, or what other difficulties you have” (HCPS3). HCPS3 also noted that ESD was not without its flaws, as the criteria can be “quite strict, and very narrow”.

#### 3.4.2 Sub-theme: informational continuity

Participants reported different ways of receiving information about the client and their care. Clinical Therapists (SLTs and OTs) report receiving community referrals directly from the hospital therapists: “all the different disciplines like physios, the OTs, SLTs, psychology, all gave a summary of what they did, and the goals that they have set out going forward. And that will be passed on then if they were being referred to community team” (HCPO13). Additional resources are often provided to the patient such as “management booklets” (HCPO13) and “home programmes” (HCPO10). HCPO10 noted that their team acknowledged the importance of making these programmes accessible for patients with communication difficulties by “giving them pictures of the exercises”. Clinical Therapists acknowledged the gaps in the information provided to them, such as “the referral onwards is on [the acute] form but it's not always filled out … that’s inconsistent, but when it is done, it is fantastic”. Additionally, HCPO10 disclosed that “I don’t think there is a section on it to put down what medications they’re on … we don’t get the hospital notes, then it has to be the patient brings it up”.

One of the Pharmacists stated that “the only information I generally get is a prescription is handed to me. I wouldn't get a massive amount of extra information beyond that” (HCPP1). Participants expressed that, given the information provided, it is difficult to discern why they are providing certain medications to the patient: “a lot of the time, we may not even know they’ve had a stroke … It’d be really handy to get even the indication … different things can have multiple indications” (HCPP2). HCPP4 considered that this information would be useful to the Pharmacist as “post-stroke they can obviously have the [impaired] swallow”, while HCPP5 noted that “there might be all these compliance aids needed”. Similar to Clinical Therapists, Pharmacists noted that information “depends on the hospital and how much they fill out on the discharge prescription. Lots of them will have spaces to say “reason for admission” or “the ward that they”re on’ and at the bottom there’d be “extra notes” or “medication that has been discontinued” and would really depend on the individual doctor if they actually fill all that out” (HCPP4). HCPP5 acknowledged that “the best-case scenario is you get a phone call from the hospital saying, “look, here”s the prescription, this person, we’ve been looking after him’, but I’d say two out of three times you don’t”, while HCPP7 also noted that these phone calls “probably happened maybe five times in 18 months”. All Pharmacists felt that the patient and their carer were their main source of information about the patient. This might pose additional barriers, as “they don’t realise that their medication has been changed or they mightn’t bring the letter to the GP so nothing gets updated or else it just gets lost in the administration” (HCPP4).

Both Pharmacists and Clinical Therapists felt that the public showed a misunderstanding of the sharing of information within the healthcare system. HCPS9 noted “once a client says it to one SLT, OT, one professional, they might not say to another person, that might be it. They might just be like sure I’ve told the SLT about my OT needs and that’s that, or I’ve told this SLT about my SLT needs so of course, the other SLT will have access to it”. HCPP5 reported the same experience, saying “A common thing is you’ll be told by the patient ‘didn’t you know? Didn’t you know I had a stroke? I thought you were all under one system’. There’s this magic system that we’re all supposed to be connected to”.

HCPS9 reported a reduced flow of information between public and private therapists: “I think for, especially some reports, it wouldn't be encouraged that a client would share it with a private company, because it's a HSE report.” Much like Pharmacists, private clinical therapists “get a lot of information from the client as well … the facilitator is that the client is knowing what their care was, and where they should be going, or who they are involved with”. HCPS8 also reported easier access to information in an acute setting as opposed to a community setting: “we know where to go if we need to find it. Like, you know the kardex is there, you know there’s a Pharmacist on the ward. You know you can chat to the nurses or one of the medical team”. Participants made suggestions regarding changes to informational continuity that they would like to see from the Irish Healthcare system moving forward. HCPS9 would like to see a “role in hospital for a discharge coordinator and have them be the person to link in with the other professionals as you go on”. HCPP7 suggested a “a fixed protocol where you can expect X amount of communication”, where “48 h prior to discharge, there’s a final assessment, like 36 h prior to discharge, the prescription is finalised and reviewed and disseminated, 24 h prior to discharge the pharmacy is contacted to give the green light that the patient will have access to what they need”. In five cases, participants called for “an integrated system” (HCPP4) where patients and providers would have access to the “[acute] care, hospital discharge service and community of care” relevant to the client (HCPS9). HCPs saw this as being “electronic” (HCPS11) or “online” (HCPP4).

#### 3.4.3 Sub-theme: relationships and relational continuity

Relationships were seen to play a central role in continuity of care by all participants. Four central relationships emerged: 1) those between HCPs, 2) those between the HCP and the patient, 3) those between the patient and their support system, and 4) those between the HCP and external organisations.

Participants were asked to describe the disciplinary approach to care post-discharge for a post-stroke patient. The majority of participants stated that the current approach to care is multidisciplinary, where “multiple disciplines [are] acting in their own silos”. HCPP7 noted that the lines between multi-, inter-, and transdisciplinary care are unclear, where “the only term that you hear in Irish healthcare is multidisciplinary. it’s like a catch all.it’s like a movable definition”. HCPS8 stated that some aspects of post-stroke care are “uni-to multi-here because [medication management]’s probably mainly resting with the Pharmacist”.

Participants acknowledged that the disciplinary approach to care may be differently structured according to the needs of the patient and the context of their care. Many participants, including HCPP7, chose to juxtapose the approach to care in the acute setting with that given in the community setting: “depending on how capable a hospital setting is, they might have interdisciplinary focus especially with stroke … In community … it definitely would be multidisciplinary and communication would be slim to none. It’ll be necessary communication only”. HCPS9 felt that public services were more likely to provide multidisciplinary care, whereas private services may operate in a more unidisciplinary manner as communication is less likely to be achieved between different companies and services.

Participants noted that core members of the MDT in community include the Social Worker, OT, SLT, General Practitioner, and Physiotherapist. In particular, “SLTs and OTs work closely together” (HCPS9). Clinical Therapists stated that “the Pharmacist doesn’t come to our multidisciplinary team meetings” (HCPS8), while Pharmacists also felt that they operate outside of the MDT: “I have never once had a contact number for a social worker, an Occupational Therapist, or a Speech and Language Therapist that would have been working with or liaising with a patient. Let’s say I noticed a problem or something, there’s absolutely nothing I can do except ask the patient to reach out to them” (HCPP7). HCPP2 noted “We aren't in that loop of information. We’re excluded from it”. HCPS9 felt this often resulted in a breakdown of care, as “a lot of clients for me haven't been able to name their SLT or their OT in public. Or I would call a hospital. And I might be like, what’s the story now? And they’re like, well, they’re discharged to community. And I might ask who’s community? And they won't know the name”.

Pharmacists most often reported liaising with the GP or contacting acute discharge staff and reported difficulty contacting acute prescribers: “[the most difficult thing]’s actually finding the same person. And secondly, is finding the relevant information. It's very, very hard. There’s no central team, you ring. Even finding the prescribers or their team can be extremely difficult. I’ve even had instances where I phoned about something and they’ve never phoned me back. Did they just forget about it? Or is it too complicated? It's very frustrating” (HCPP6).

HCPS3 emphasised the benefit of professional relationships between healthcare providers and social supports in the community, with reference to the Stroke Association (Stroke.org.uk, 2023), Aphasia Café (UCC.ie, 2023) and communication groups. They noted that this is particularly important for “patients who say they don’t have very severe difficulties, but there’s still something going on … sometimes having peer support and attending group sessions … can be just as beneficial, if not more beneficial sometimes to patients”. HCPS9 considered that this benefit came in the form of “hearing information from peers … to also link in with and contact and offer to keep up that continuity of care”.

While the relationship between the patient and the healthcare provider was viewed as an important component of continuity of care, participants found this to be the most open to communicative breakdowns. Participants attributed this mostly to the inconsistency of HCPs involved in the patient’s care: “I think it can change a lot. I think that can be really confusing” (HCPO10). HCPS9 encountered patients who “often say that, ‘oh, another person’s changed. It's another person, it's another person the whole time’ and that does impact the therapeutic relationship”. HCPS9 attributed this to “a mix of things in terms of the workers themselves wanting to move places, they could feel burnt out, the resources there, but it's also the lack of permanent positions they might be able to get”, while HCPO10 regarded this as a more systemic issue “they’ll have an OT in the hospital, then they’ll have an OT in ESD, let’s say they have a primary care need, that will be a separate person. And then if they come to community rehab, OT rehab, that will be another person again”. HCPO14 relayed the story of a patient who felt that “every time I go in, it’s a different one and I end up telling the story all over again and they don’t know me and they don’t really care about me because I’m just in there for 15-min appointments”. HCPO12 felt “that the client can’t build a relationship with their doctor, and then they’re not inclined to kind of tell them what’s really going on”. However, HCPS8 felt that “people should be able to move around easier in their employment … the methodology is described and the pathway is there. So I wouldn't be too concerned about [staff rotating], I think it's a healthy thing”. HCPS3 and HCPS9 both felt that an outreach service would address this inconsistency, “so they get picked up quickly and they’re seeing a familiar face”.

Participants reported that the relationship between the patient and their individual support systems were great facilitators to continuity of care. Participants described these systems as including family and friends, carers, and home help. HCPP6 noted that “The carers are usually very good … [patients] just want to get on with things and just somebody to look after them”. Participants saw the carer as playing a prominent role in helping to manage medications, organise appointments, and to advocate for the patient post-stroke. Pharmacists saw carers as an important point of contact between them and the other members of the MDT. HCPO13 spoke of the importance of providing carers with exercises between blocks of care and “setting them up with [a handover], having the information concise for their carers or family that are continuing the patient’s care when they go home”.

## 4 Discussion

Several existing studies have examined the relationship between continuity of care and medication adherence in patients with chronic diseases (Kerse et al., 2004; Robles and Anderson, 2011; Chen et al., 2013). However, a majority of this research examines the relationship quantitatively (Warren et al., 2015; Dossa et al., 2017). Participants recognised the importance of continuous care for the patient post-discharge, and acknowledged the central role that this may play in medication non-adherence. However, it was observed that HCPs view medication adherence as a multifactorial issue, of which continuity of care is only one aspect. This reflects findings from qualitative studies of HCPs conducted by Kvarnström et al. (Kvarnström et al., 2018) and Jaam et al., (Jaam et al., 2018).

The factors influencing medication adherence as identified by participants in this study broadly correspond with the WHO’s Multidimensional Adherence Model (AlGhurair et al., 2012). This ecological model considers intra- and interpersonal, systemic, regulatory, and community barriers to medication adherence (AlGhurair et al., 2012). The results of this study show that the boundaries between these influencing factors are not always clear. Often patient-related and illness- or condition-related factors were found to overlap, with broad terms such as “understanding” used in relation to both. This may reflect not only the complexity of influencing factors for medication adherence, but a lack of separation found between the patient and their condition (Karnilowicz, 2011). Stroke survivors often feel a loss of identity following their CVA, as stroke may impair not only their abilities, but their resources to scaffold their recovery. Healthcare providers highlighted a lack of patient desire to increase knowledge as a patient-related factor impacting medication adherence—however, linguistic, or cognitive deficits may be a barrier to the knowledge needed to understand their medication regimen properly. The unification of these factors may lead to inadequate interventions which do not fully address the root of the issue. A study by Alfian et al., 2020 found that sociodemographic and clinical factors were not associated with non-adherence to antihypertensive drugs, while higher necessity beliefs were associated with less non-adherence. Al-Lawati, 2014 showed that regular interactions between patients and their healthcare providers result in higher adherence rates for all patients. Therefore, patient education programmes designed to convey the importance of treatment may be highly effective, particularly when delivered routinely, and with consistency across members of the multidisciplinary team.

Patient education programmes tend to be more successful when the preventative counselling is interactive and the HCP has appropriate access to resources (e.g., time available to HCPs, suitable counselling materials, knowledge, and skills). This underscores the need for training for healthcare staff to ensure high-quality counselling and patients' adherence to secondary preventative behaviours (Oikarinen et al., 2017). HCPs involved in this study expressed a desire for this training to have an interprofessional focus, whether this be conducted during third-level education or continuous professional development. While participants in this study indicate a knowledge of the importance of consistent patient counselling and display a willingness to conduct these sessions, constraints on time, knowledge of interdisciplinary roles, and access to resources impede their ability to do so.

Participants have shown a desire for the appointment of a Stroke Key Worker, whose role would be to provide specific support and advice to stroke patients and their families, and to assist with the transition of care from hospital to home. The Irish National Stroke Strategy 2022–2027 (Health Service Executive, 2022) has outlined its intention to appoint one such Key Worker in each community health organisation, with the role being piloted in a single site in 2023. While current research has shown the benefit of stroke co-ordinators (Fisher et al., 2021; Hitch et al., 2020; von Koch et al., 2000), future research should aim to evaluate the effectiveness of this role within an Irish context.

The Irish National Stroke Strategy also outlines its intent to increase ESD sites. HCPs in this study criticised the narrow inclusion criteria of these ESD services, which was highlighted in a recent Cochrane review that revealed a median of only 33% of patients met the inclusion criteria for ESD programmes (Langhorne et al., 2017). However, the Irish government aims to increase the number of ESD sites from the current nine to twenty-one by the end of 2025 (Health Service Executive, 2022). This increase in sites may allow the service to work under less narrow criteria. Participants in this study praised the ESD service for its contributions to continuity of care and patient re-integration in the community.

One benefit of ESD in comparison to usual stroke care is the inclusion of a well-defined team structure. Participants in this study reported difficulties in identifying, knowing the roles of, and establishing lines of communication with community HCPs. Pharmacists reported feeling more separated from other members of the MDT than SLT or OT. This aligns with findings from a study by Weiss et al., 2014 who found that 22% of Pharmacists did not consider themselves to be part of a multidisciplinary team. This same study found that only 1% of Pharmacists reported working with SLTs on a regular basis, while 3% of Pharmacists reported working with OTs on a regular basis. Regular communication and a positive working relationship with the multidisciplinary team are considered crucial for increasing medication adherence for those with chronic conditions (Herrerias et al., 2022).

Written communication was noted as an important method of communication between HCPs and patients in this study. The Irish National Stroke Strategy (Health Service Executive, 2022) proposes the introduction of a stroke passport, in addition to the current discharge report. This stroke passport will allow the patient to maintain accurate and timely records of their care and assistance throughout their rehabilitation. Participants report using similar, informal strategies in their current practice, however noted that current discharge reports are often not completed to the highest standard. It is fair to assume that the addition of a further discharge document will increase workload and therefore also may not be completed satisfactorily. Though the Health Research and Quality Authority (HIQA) have published a National Standard for Patient Discharge Summary Information (Health Research and Quality Authority, 2013), it was found in 2019 that the standard of discharge summaries from secondary care still fell short of accepted standards (O'Connor et al., 2019). Future research should examine whether the suggested interventions have been successful at improving discharge report standards and determine the persistent areas of concern in order to best facilitate informational continuity.

## 5 Limitations

A greater number of Pharmacists participated in the study than SLTs or OTs, which may have influenced the findings. However, this may simply be reflective of HCPs’ views of the more prominent role that the Pharmacist plays in the management of medication adherence post-stroke. Similarly, thirteen of the fourteen participants in this study were female. However, this reflects the current gender imbalance among HCPs in Ireland, particularly in the fields of SLT and OT.

As participant recruitment was carried out through word-of-mouth, this may have introduced sampling bias by limiting the reach of the project. This may also have limited the diversity of the participant pool.

## 6 Conclusion

This study explored the knowledge, attitudes, and beliefs of HCPs regarding continuity of care post discharge from stroke wards in Ireland, and its impact on medication adherence post-stroke. HCP participants reported that additional resources must be provided in order to bring community healthcare to the same standard currently provided by acute care. Increased channels of communication must be established across contexts and disciplines. While suboptimal continuity of care was reported as contributing to medication non-adherence, HCPs also acknowledged the complexities of medication management for the patient post-stroke.

## Data Availability

The raw data supporting the conclusion of this article will be made available by the authors, without undue reservation.
