# Supplementary material for: Healthcare professional perspectives on medication challenges in the post-stroke patient
Source: Front Pharmacol. 2023 Nov 21;14:1266277. doi: 10.3389/fphar.2023.1266277 (PMC10702955; doi:10.3389/fphar.2023.1266277)
Supplement: Supplementary file 1 [file DataSheet1.docx]

**Appendix I** – HCPs Semi-Structured Interview Topic Guide

- What is your professional background? How long have you been practicing?
- What would indicate that you are dealing with post-stroke patient? What is the profile of typical post-stroke patient?
- How would you define the term “continuity of care”?
- What does this look like in terms of a patient who has been discharged from the stroke ward?
- What do you see as the greatest barriers to continuity of care post-discharge from the stroke ward?
- What do you see as the greatest facilitators to continuity of care post-discharge from the stroke ward?
- In your experience, is care provided to the post-stroke patient by the same central providers?
- Do you feel you/ your team have the knowledge and resources to adapt care to stroke patients’ behavioural, personal, and cultural beliefs? Can you provide an example of this?
- How involved is the patient in the planning of care and shared-decision making post-discharge from stroke ward?
- Are you typically aware of other HCPs the client has been referred to post-discharge? How are you made aware of the client’s involvement with these HCPs?
- Considering medication, what information is typically communicated to you about the client? How is this communicated to you? Do you find this information to be sufficient? Why/ why not?
- Do you find that post-stroke patients are typically knowledgeable about their medications?
- What do you think are the barriers to medication adherence are post-stroke?
- What do you think are the facilitators to medication adherence are post-stroke?
- Which HCPs should be involved in medication adherence post-stroke? Are these HCPs currently involved, in your experience?
- Do you believe discharge information relating to medication is received in a timely manner? What are the barriers/ facilitators to receiving this information in a timely manner?
- With regard to the post-stroke patient, do you feel the approach to management post-discharge is unidisciplinary, multidisciplinary, inter-disciplinary, trans-disciplinary? Are these boundaries clear?
- What are your thoughts on the management of the transition of care from hospital to home for a patient post-stroke? What works well? What areas could be improved?
